# Supplementary material for: Parameter optimisation for mitigating somatosensory confounds during transcranial ultrasonic stimulation
Source: bioRxiv. 2025 Mar 19:2025.03.19.642045. Preprint. [Version 1] doi: 10.1101/2025.03.19.642045 (PMC11956992; doi:10.1101/2025.03.19.642045)
Supplement: 1 [file NIHPP2025.03.19.642045V1-supplement-1.pdf]

bioRxiv preprint doi: https://doi.org/10.1101/2025.03.19.642045; this version posted March 19, 2025. The copyright holder for this preprint (which was not certified by peer review) is the author/funder, who has granted bioRxiv a license to display the preprint in perpetuity. It is made available under aCC-BY 4.0 International license.

Supplementary Material

Table of Contents

Supplementary Material **1**

Supplementary Table 1 | TUS specifications per ITRUSST standardised reporting guidelines **2**

Supplementary Table 2 | MRI acquisition parameters **5**

Supplementary Table 3 | Post-hoc pairwise comparisons for the effect of PRF on thresholds **6**

Supplementary Fig. 1 | Full-field hydrophone measurements **7**

Supplementary Fig. 2 | Near-field hydrophone measurements **8**

Supplementary Fig. 3 | Simulations **10**

Supplementary Fig. 4 | Thresholding procedure **11**

Supplementary Fig. 5 | Study procedure and counterbalancing **13**

Supplementary Fig. 6. | VAS responses to sham trials **16**

Supplementary Fig. 7 | Co-occurrence of somatosensory percepts **17**

Supplementary Fig. 8 | Dose-response (500 kHz) **18**

Supplementary Fig. 9 | Inter-trial temporal summation **19**

References **20**

Supplementary Table 1 | TUS specifications per ITRUSST standardised reporting[1] guidelines

Transducer and drive system parameters

Transducer	Centre frequency	Radius of curvature	Aperture diameter	Number of elements	Element distribution	Matching	Drive system
250-2CH*	250 kHz	45 mm	45 mm	2	annular array, bowl, equal area	2-channel electrical impedance matching network using a 4-to-2 combination network	TPO-203-035
500-2CH*	500 kHz	45 mm	45 mm	2	annular array, bowl, equal area	2-channel electrical impedance matching network	TPO-105-010
250-4CH*	250 kHz	64 mm	64 mm	4	annular array, bowl, equal area	4-channel electrical impedance matching network	TPO-105-010

*Manufacturer: Sonic Concepts Inc., Bothell, WA; Supplier/support: BrainBox Ltd., Cardiff, UK.

Driving system settings

Transducer	Operating frequency	TPO output I_{SPPA} setting	Measured I_{SPPA}*	Measured $I_{SPPA.SCALP}$*	Focal position setting
250-2CH	250 kHz	12.5 W/cm^2	9.86 W/cm^2	6.53 W/cm^2	35.7 mm
		25 W/cm^2	19.72 W/cm^2	13.06 W/cm^2	38.3 mm
		37.5 W/cm^2	29.59 W/cm^2	19.59 W/cm^2	40.3 mm
		50 W/cm^2	39.45 W/cm^2	26.13 W/cm^2	42.1 mm
					44.1 mm
500-2CH	500 kHz	30 W/cm^2	32.57 W/cm^2	18.47 W/cm^2	33.2 mm
		50 W/cm^2	54.29 W/cm^2	30.78 W/cm^2	
		70 W/cm^2	76.00 W/cm^2	43.09 W/cm^2	
250-4CH	250 kHz	33.1 W/cm^2	28.94 W/cm^2	13.82 W/cm^2	30.4 mm

*Hydrophone measurements were made to determine the actual I_{SPPA} value in free-water. These values are used throughout the article.

Free field pressure parameters

Transducer	Measured field	I_{SPPA} (W/cm²)*	Position of I_{SPPA} (mm)**	volume -3dB (mm³)	lateral -3dB (mm)	axial -3dB (mm)	volume -6dB (mm)	lateral -6dB (mm)	axial -6dB (mm)
250-2CH	full	23.669	37	745.5	5.59	31.48	2771.25	8.33	52.79
	near	15.676	9.5	51.22	3.07	7.05			
500-2CH	full	32.574	33.5	152.5	3.47	17.65	551.25	5.23	29.17
	near	18.468	6.75	11.64	1.97	3.96			
250-4CH	full	26.229	28	223.25	3.85	19.05	770.63	5.41	35.79
	near	12.527	12.5	31.19	2.06	13.48			

*The I_{SPPA} was calculated for a TPO interface I_{SPPA} setting of 30 W/cm². I_{SPPA} for the full-field measurement is relevant to the focal region in the brain, while I_{SPPA} for the near-field measurement refers to the peak-intensity in the near-field relevant for the scalp. This is referred to as $I_{SPPA.SCALP}$ in the main text.
**The axial position of the I_{SPPA} relative to the exit plane of the transducer.

Upper-bound safety metrics

Transducer	Max. free-water I_{SPPA}	$I_{SPPA\_TC\_SIM}$ (%transmission)[1]	MI_{TC_SIM}[2]	MI_{TC_EST}	Max. $I_{SPPA.SCALP}$	MI_{SCALP_EST}[4]	Max. TR[5]	CEM 43°C[6]
250-2CH	39.45 W/cm²	17.1 W/cm² (43.5%)	1.43	1.62	26.13 W/cm²	1.83	0.95 °C	1.17e-05
500-2CH	76.00 W/cm²	26.8 W/cm² (35.3%)	1.27	1.33	43.09 W/cm²	1.66	1.07 °C	1.09e-05

Safety metrics are calculated for upper-bound stimulation parameters (i.e., highest doses) to demonstrate the safety of all protocols administered during this study.
[1]Representative simulated transcranial I_{SPPA} in the brain.
[2]Representative simulated transcranial mechanical index (MI_{TC_SIM}) in the brain.
[3]Estimated transcranial mechanical index (MI_{TC_EST}) using the open-source TUS calculator: https://www.socsci.ru.nl/fusinitiative/tuscalculator/.
[4]Estimated mechanical index in the scalp (MI_{SCALP_EST}) using the open-source TUS calculator: https://www.socsci.ru.nl/fusinitiative/tuscalculator/.
[5]Maximum simulated thermal rise (TR). The simulation was run for the highest dose level for both transducers.
[6]Thermal dose in cumulative equivalent minutes (CEM) at 43 °C.
All parameters fall within ITRUSST biophysical safety recommendations[2].

Pulse timing parameters

Protocol types		Duration	Ramp shape	Ramp duration	Repetition interval (frequency)
**standard**/*'dose modality: pulse duration'*	pulse	*50/**100**/150/200 ms*	**none**	**none**	**200ms (5Hz)**
	pulse train	**1 s**	**none**	**none**	
'temporal summation: longer PTD'	pulse	100 ms	none	none	200ms (5Hz)
	pulse train	*10 s*	none	none	
'ramping'	pulse	100 ms	*Tukey*	*0/1/5/50 ms*	200ms (5Hz)
	pulse train	1 s	none	none	
'PRF'	pulse	*1/2/5/10/100/200 ms*	*Tukey*	*0.5/1/2.5/5/50/ 100 ms*	*1ms (1000Hz), 2ms (500Hz), 5ms (200 Hz), 10ms (100Hz), 100ms (10Hz), 200ms (5Hz)*
	pulse train	1 s	none		

This table depicts the pulse timing parameters for the standard protocol in **blue**, as well as the relevant timing for manipulated parameters in *italics*. This includes the investigation of dose by varying pulse duration, the longer pulse train duration (PTD) applied to mimic offline protocols while participants continuously rated somatosensory co-stimulation, ramping, and pulse repetition frequency (PRF) where full ramping was administered.

bioRxiv preprint doi: https://doi.org/10.1101/2025.03.19.642045; this version posted March 19, 2025. The copyright holder for this preprint (which was not certified by peer review) is the author/funder, who has granted bioRxiv a license to display the preprint in perpetuity. It is made available under aCC-BY 4.0 International license.

Supplementary Table 2 | MRI acquisition parameters

Scan	TR	TE	FoV read	FoV phase	voxel	N slices
T1w	2700 ms	3.69 ms	230 mm	128.1%	0.9 mm iso	224
UTE	3.6 ms	0.07 ms	240 mm	100.0%	0.8 mm iso	320

This table shows the MRI acquisition parameters. Scans were acquired using a 3T Siemens Skyra MRI scanner (Siemens Medical Solutions, Erlangen, Germany) with a 32-channel head coil. Anatomical T1w scans were used for online neuronavigation. UTE scans were acquired to generate pseudo-CT images for post-hoc simulation

bioRxiv preprint doi: https://doi.org/10.1101/2025.03.19.642045; this version posted March 19, 2025. The copyright holder for this preprint (which was not certified by peer review) is the author/funder, who has granted bioRxiv a license to display the preprint in perpetuity. It is made available under aCC-BY 4.0 International license.

Supplementary Table 3 | Post-hoc pairwise comparisons for the effect of PRF on thresholds

Contrast	p value (uncorrected)	p value (FDR corrected)
PRF5 - PRF10	0.4893	0.6146
PRF5 - PRF50	0.9895	0.9895
PRF5 - PRF100	0.8800	0.9240
PRF5 - PRF200	0.0006*	0.0042*
PRF5 - PRF500	0.0446*	0.0967.
PRF5 - PRF1000	0.0208*	0.0645.
PRF10 - PRF50	0.4976	0.6146
PRF10 - PRF100	0.3998	0.5597
PRF10 - PRF200	0.0054*	0.0283*
PRF10 - PRF500	0.1848	0.2986
PRF10 - PRF1000	0.1023*	0.1952
PRF50 - PRF100	0.8696	0.9240
PRF50 - PRF200	0.0006*	0.0042*
PRF50 - PRF500	0.0460*	0.0967.
PRF50 - PRF1000	0.0215*	0.0645.
PRF100 - PRF200	0.0003*	0.0042*
PRF100 - PRF500	0.0311*	0.0817.
PRF100 - PRF1000	0.0139*	0.0586.
PRF200 - PRF500	0.1375	0.2406
PRF200 - PRF1000	0.2393	0.3590
PRF500 - PRF1000	0.7555	0.8814

Post-hoc paired comparisons between each applied level of PRF, both without correction for multiple comparison (middle column), and with false discovery rate (FDR) correction for multiple comparisons. '*' = significant, '.' = trend.

bioRxiv preprint doi: https://doi.org/10.1101/2025.03.19.642045; this version posted March 19, 2025. The copyright holder for this preprint (which was not certified by peer review) is the author/funder, who has granted bioRxiv a license to display the preprint in perpetuity. It is made available under aCC-BY 4.0 International license.

Supplementary Fig. 1 | Full-field hydrophone measurements

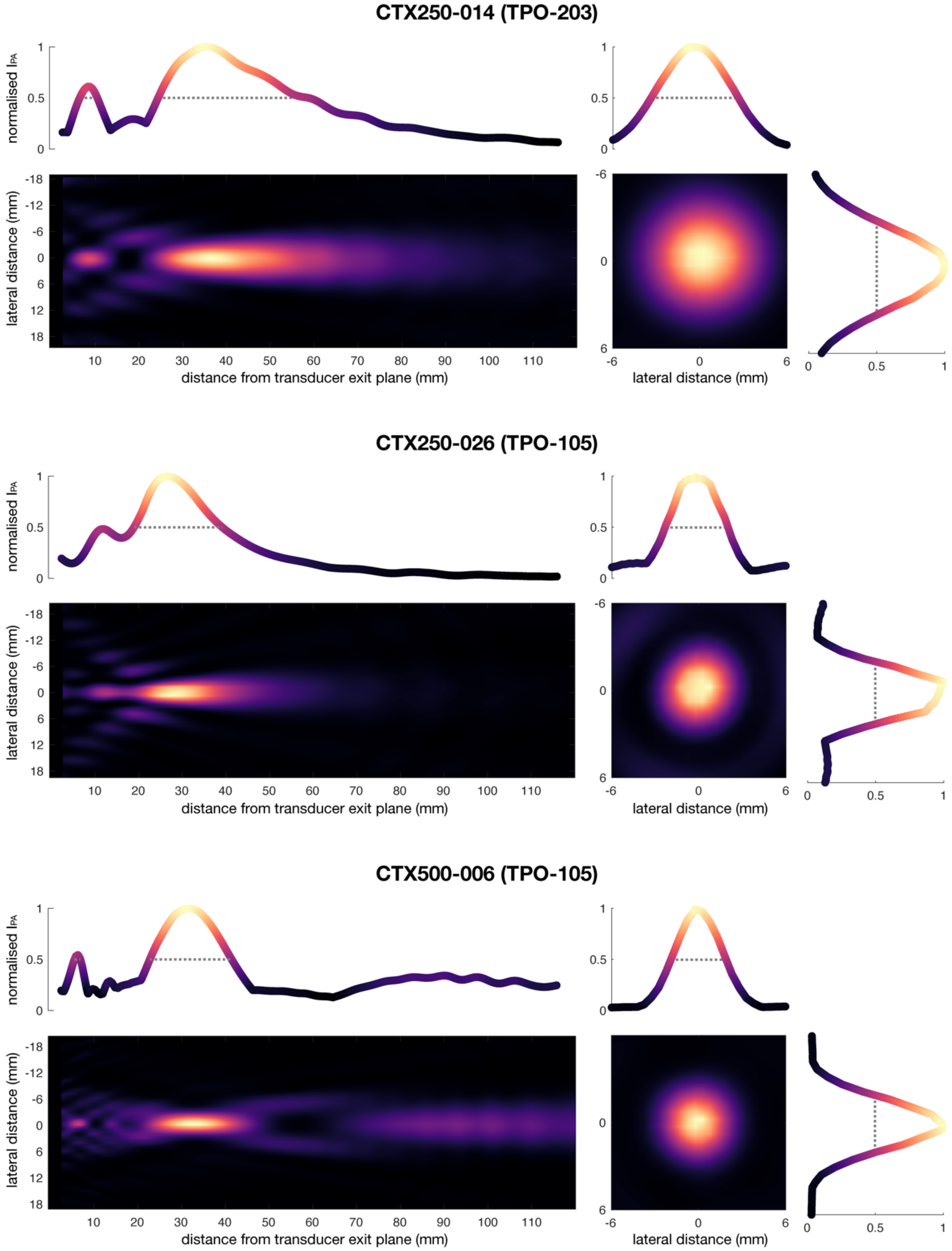

Hydrophone measurements of the min-max normalised pulse-average intensity (I_{PA}) for the full acoustic field along the axial plane (left) and the lateral cross-section at the focus (right) of each transducer. The original resolution (0.5 mm) has been upscaled for visualisation by a factor of 10 using linear interpolation. Intensity distribution lines depict the maximum I_{PA} per slice, where the full-width-half-maximum of the I_{SPPA} (FWHM; -3dB) is indicated by the dotted grey line.

bioRxiv preprint doi: https://doi.org/10.1101/2025.03.19.642045; this version posted March 19, 2025. The copyright holder for this preprint (which was not certified by peer review) is the author/funder, who has granted bioRxiv a license to display the preprint in perpetuity. It is made available under aCC-BY 4.0 International license.

Supplementary Fig. 2 | Near-field hydrophone measurements

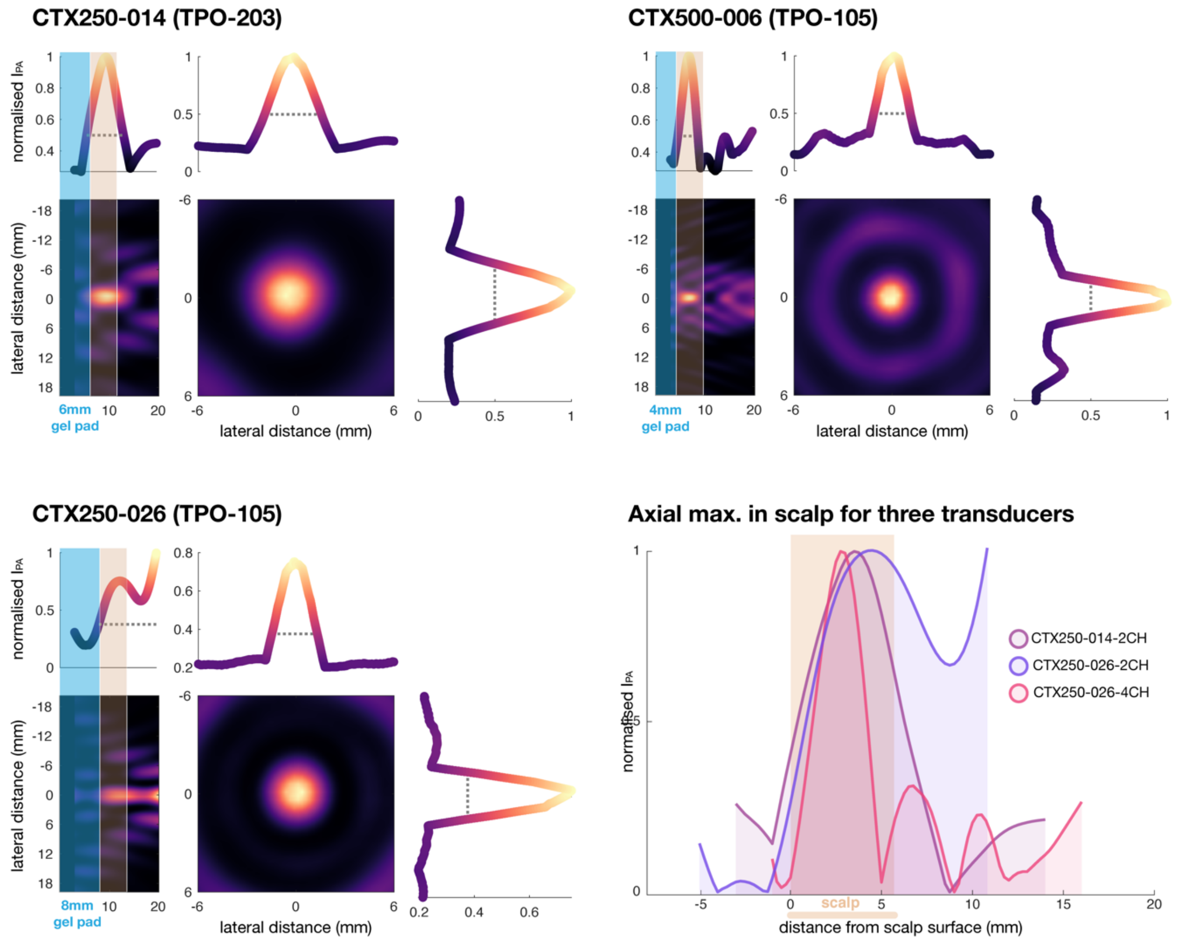

Hydrophone measurements of the min-max normalised pulse-average intensity (I_{PA}) for the near-field at an increased resolution of 0.25 mm. These data were used to determine the gel pad thicknesses (blue) and stimulation intensities. Note that the bottom-right panel depicts the normalised distributions of the axial maximum intensity for each transducer. In practice, the relative stimulation intensities of the transducers were adjusted to achieve comparable levels of integrated maximum intensity and integrated total intensity in the scalp (beige) between transducers (see main text Fig. 4).

Near-field hydrophone measurements were required to accurately assess the effects of fundamental frequency and transducer aperture diameter on peripheral somatosensory co-stimulation. These investigations involved different transducers with varying intensity profiles, which had to be equalised to make valid comparisons.

To this end, we utilised the integrated maximum and/or total intensity in the scalp (5.5 mm width[3–5]) as a metric to optimise comparability between transducers. First, we identified focal depth settings at which the acoustic profiles were most similar (250-2CH: 40.3 mm; 250-4CH: 30.4 mm; 500-2CH: 33.1 mm). Next, we determined the gel pad thicknesses to optimise coherence of acoustic profiles and the integrated maximum and/or total intensity in the scalp (250-2CH: 6 mm; 250-4CH: 8 mm; 500-2CH: 4 mm). Finally, we set stimulation intensities in the scalp. For equal integrated maximum intensities, we compared fundamental frequencies using transducer 250-2CH at 26.13 W/cm^2 and transducer 500-2CH at 30.78 W/cm^2 $I_{SPPA.SCALP}$. For equal integrated total intensity, the intensity for 500-2CH was increased to 43.09 W/cm^2. The intensities

bioRxiv preprint doi: https://doi.org/10.1101/2025.03.19.642045; this version posted March 19, 2025. The copyright holder for this preprint (which was not certified by peer review) is the author/funder, who has granted bioRxiv a license to display the preprint in perpetuity. It is made available under aCC-BY 4.0 International license.

used to compare transducer aperture diameter between 250-2CH and 250-4CH were 13.06 W/cm^2 and 13.82 W/cm^2 $I_{SPPA.SCALP}$, respectively.

Hydrophone measurements were performed using an independent metrology setup enabling accurate positioning of a calibrated hydrophone ($d_{x,y,z}$ = 5 μm; HGL 0200, Onda Corp., Sunnyvale, USA). The transducer was submerged in degassed, filtered, and deionised water at ambient temperature in a plexiglass water tank (150x200x400 mm). A custom probe holder ensured orthogonal alignment of the transducer and hydrophone.

The transducer was set to deliver 250 μs square-wave pulses at a power of 2.5 or 5.0 W per channel. These pulses were registered using a PicoScope 5244D (Pico Technology, UK) at a sampling frequency of 25 MHz using a custom closed-loop control program triggered by the transducer power output system.

For full-field measurements, line scans were performed with 0.5 mm steps along the beam axis, centred on the focus, at distances from 3 to 120 mm relative to the exit plane of the transducer. A ~38 mm range was measured across the lateral cross-sections of the ultrasound beam. Full-field measurements were acquired to inform transcranial ultrasound for the experiment.

To capture a higher resolution intensity field for depths relevant to peripheral stimulation of the scalp, we recorded near-field intensities using 0.25 mm steps for an axial range of 3 to 20 mm from the transducer exit plane, and a lateral range of ~40 mm. Near-field measurements were acquired to inform the gel pad thicknesses and absolute free-water intensities required to equalise the integrated maximum intensity and/or integrated total intensity in the scalp.

Post-processing involved an FFT-based method to acquire a single average amplitude reading for each recorded pulse over the window between the pulse ring-up time and pulse cessation. Subsequently, the complete pressure field was spatially filtered along the axial direction using a FIR Butterworth low-pass filter to remove oscillating interference caused by reflections off the hydrophone.

The measured intensities were then re-scaled using the factor $Power_{experiment}/Power_{hydrophone.measurement}$. Next, the location and value of the spatial-peak pulse-average intensity at the focus (I_{SPPA}) and the peak near-field intensity ($I_{SPPA.SCALP}$) were extracted and the focal dimensions of the -3dB focal region were calculated using in the *regionprops3* MATLAB function.

Both full- and near-field measurements were performed for three transducers (i.e., 250-2CH – TPO-203, 500-2CH – TPO-105, and 250-4CH – TPO-105; see Supplementary Table 2 for detailed specifications), at focal depth settings of 40.3, 33.2, and 30.4 mm, respectively.

bioRxiv preprint doi: https://doi.org/10.1101/2025.03.19.642045; this version posted March 19, 2025. The copyright holder for this preprint (which was not certified by peer review) is the author/funder, who has granted bioRxiv a license to display the preprint in perpetuity. It is made available under aCC-BY 4.0 International license.

Supplementary Fig. 3 | Simulations

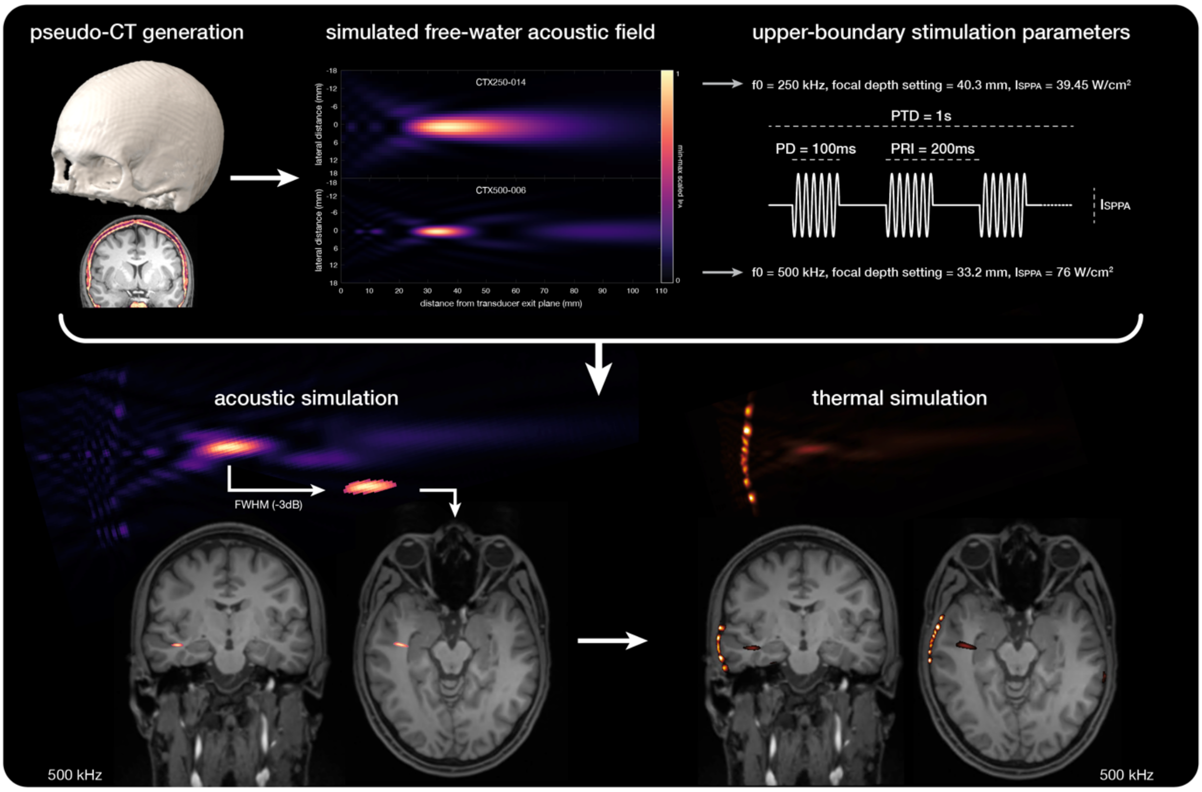

Pseudo-CT scans (top left) were generated from UTE scans and used to assign acoustic medium properties. Next, we confirmed that the free-field simulations for both 250-2CH and 500-2CH transducers corresponded well with hydrophone measurements. We then ran acoustic and thermal simulations for upper-bound stimulation parameters to obtain safety-relevant metrics (top right). The transcranial full-width half-maximum (FWHM) intensity for 500 kHz stimulation is depicted on the bottom left. The subsequent thermal simulation is depicted on the right.

We ran a representative simulation of acoustic wave propagation for 250 kHz and 500 kHz TUS using k-Plan, a user interface for the pseudo-spectral time-domain solver k-Wave[6]. First, we generated a pseudo-CT (pCT) scan from our ultra-short echo time (UTE) MRI scan using the open-source 'petra-to-pct' toolbox (https://github.com/ucl-bug/petra-to-ct)[7]. Histogram normalisation was set to two peaks at a minimum distance of 1000 units and skull mask smoothing was set to 5 mm.

In k-Plan, we first simulated our custom transducer models in free-water and confirmed that the full-field intensity profile was comparable to our hydrophone measurements. Next, we ran acoustic and thermal simulations to assess acoustic targeting and to estimate upper-boundary safety metrics for these transducers. The maximum dose was simulated with: PD = 100 ms, PRI = 200 ms, PTD = 1 s, $I_{SPPA.FREE.WATER}$ = 39.45 W/cm^2 (250 kHz) or 76 W/cm^2 (500 kHz).

The simulated pressure field was exported using the 'k-plan-matlab-tools' toolbox (https://github.com/ucl-bug/k-plan-matlab-tools). In MATLAB, the intensity field was calculated using $I = \frac{P^2}{2\rho c}$, where ρ was 1000 kg/m^3 and c was 1500 m/s. A full-width half-maximum threshold (-3dB) was then applied to the intensity field, and the resulting field was overlaid onto the MRI scan.

bioRxiv preprint doi: https://doi.org/10.1101/2025.03.19.642045; this version posted March 19, 2025. The copyright holder for this preprint (which was not certified by peer review) is the author/funder, who has granted bioRxiv a license to display the preprint in perpetuity. It is made available under aCC-BY 4.0 International license.

Supplementary Fig. 4 | Thresholding procedure

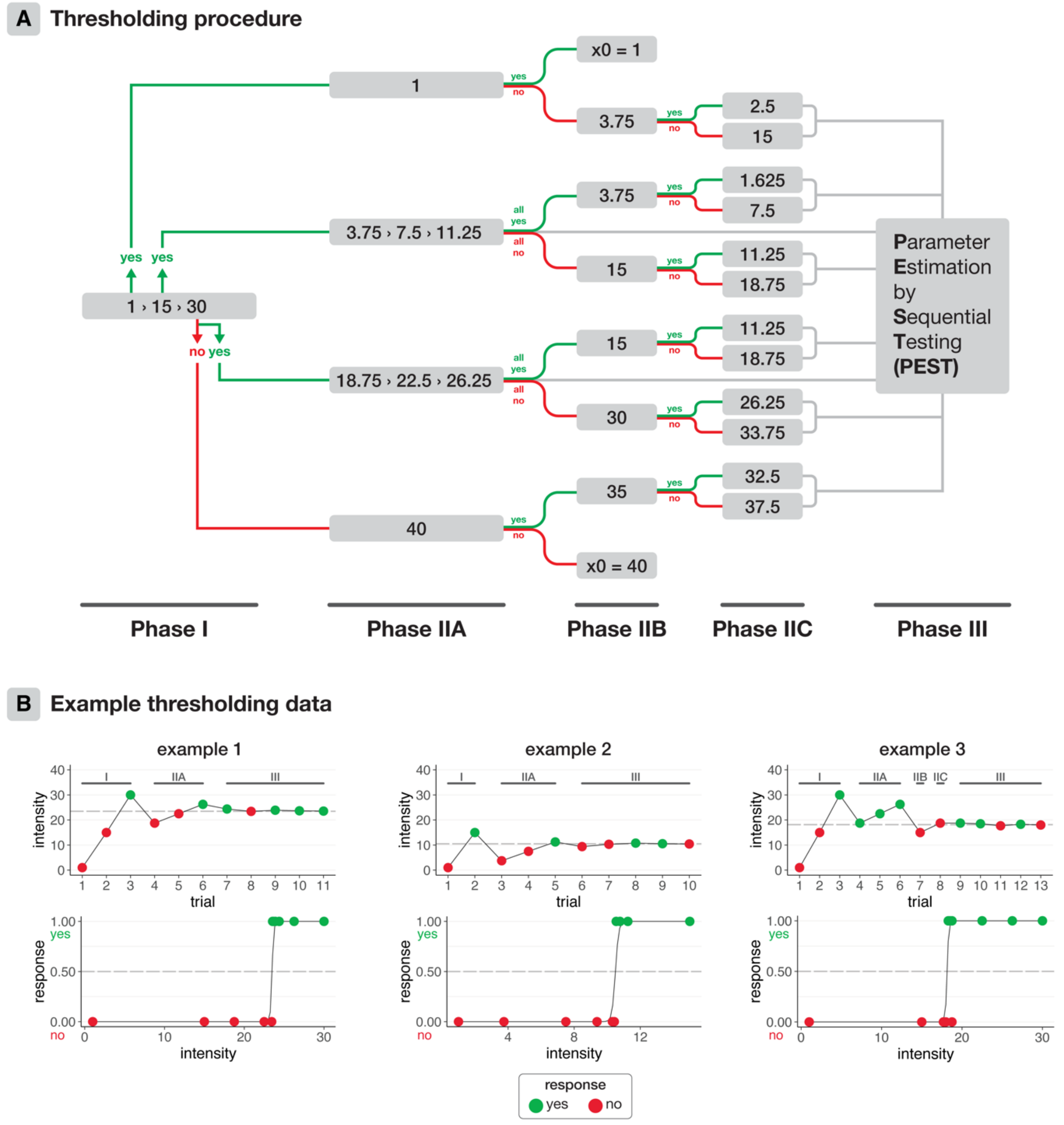

(A) Flowchart of the custom thresholding procedure. **(B)** Example thresholding data. For the top panels, the TPO interface I_{SPPA} is displayed across trials to demonstrate the operation of the thresholding procedure. Green and red dots indicate 'yes' and 'no' responses respectively to whether the stimulus was felt. The dotted grey line depicts the estimated sensory threshold. The bottom panel depicts the fitted psychometric curve over the binary yes/no responses after completion of the thresholding procedure. In examples 1 and 2, the participant transitioned between 'no' and 'yes' within the three intermediate stimulus intensities tested in Phase IIA, therefore continuing directly to Parameter Estimation by Sequential Testing (PEST; Phase III). In example 3, the participant responded yes to all three intermediate intensities, so the lower boundary was re-tested (Phase IIB). Since the response was 'no', a slightly higher boundary was tested (Phase IIC). Then, the thresholding procedure continued to Phase III.

bioRxiv preprint doi: https://doi.org/10.1101/2025.03.19.642045; this version posted March 19, 2025. The copyright holder for this preprint (which was not certified by peer review) is the author/funder, who has granted bioRxiv a license to display the preprint in perpetuity. It is made available under aCC-BY 4.0 International license.

We measured sensory thresholds to precisely capture the effects of pulse repetition frequency and ramping on somatosensory co-stimulation. Typically, many trials are required to estimate sensory thresholds[8]. In the present experiment, that would have required a prohibitively large amount of ultrasonic stimulation. Therefore, we designed a custom thresholding procedure that consisted of three phases (Supplementary Fig. 4A).

Phase III consisted of five trials where we iteratively fit a logistic, psychometric function to the binary response data of all preceding trials using a Parameter Estimation by Sequential Testing (PEST) method[9,10]. We defined the logistic function as:

$$P(I) = \frac{1}{1 + e^{-k(I - x0)}}$$

where I is the TPO interface I_{SPPA} value, $x0$ is the stimulus intensity at which the detection probability is an estimated 50%, and k is the slope of the psychometric curve. The curve was fit using the 'curve_fit' function from the SciPy package in Python. Initial parameter values for $x0$ and k were set to the *t-1* stimulus intensity and 1, respectively, to improve convergence. We constrained the optimisation with bounds of -5-40 for $x0$ and 0.01-100 for k.

In some cases, participants already reported feeling stimulation at a TPO intensity setting of 1 W/cm^2, or didn't report feeling anything from 1-30 W/cm^2. In the prior case, Phase IIA re-tested this minimum stimulus intensity. If the participant continued to respond 'yes', 1 W/cm^2 was set as their threshold (floor effect). When participants did not report feeling anything from 1-30 W/cm^2, in Phase IIA stimulation intensity was increased to 40 W/cm^2. If participants still did not feel anything, 40 W/cm^2 was set as their threshold (ceiling effect).

bioRxiv preprint doi: https://doi.org/10.1101/2025.03.19.642045; this version posted March 19, 2025. The copyright holder for this preprint (which was not certified by peer review) is the author/funder, who has granted bioRxiv a license to display the preprint in perpetuity. It is made available under aCC-BY 4.0 International license.

Supplementary Fig. 5 | Study procedure and counterbalancing

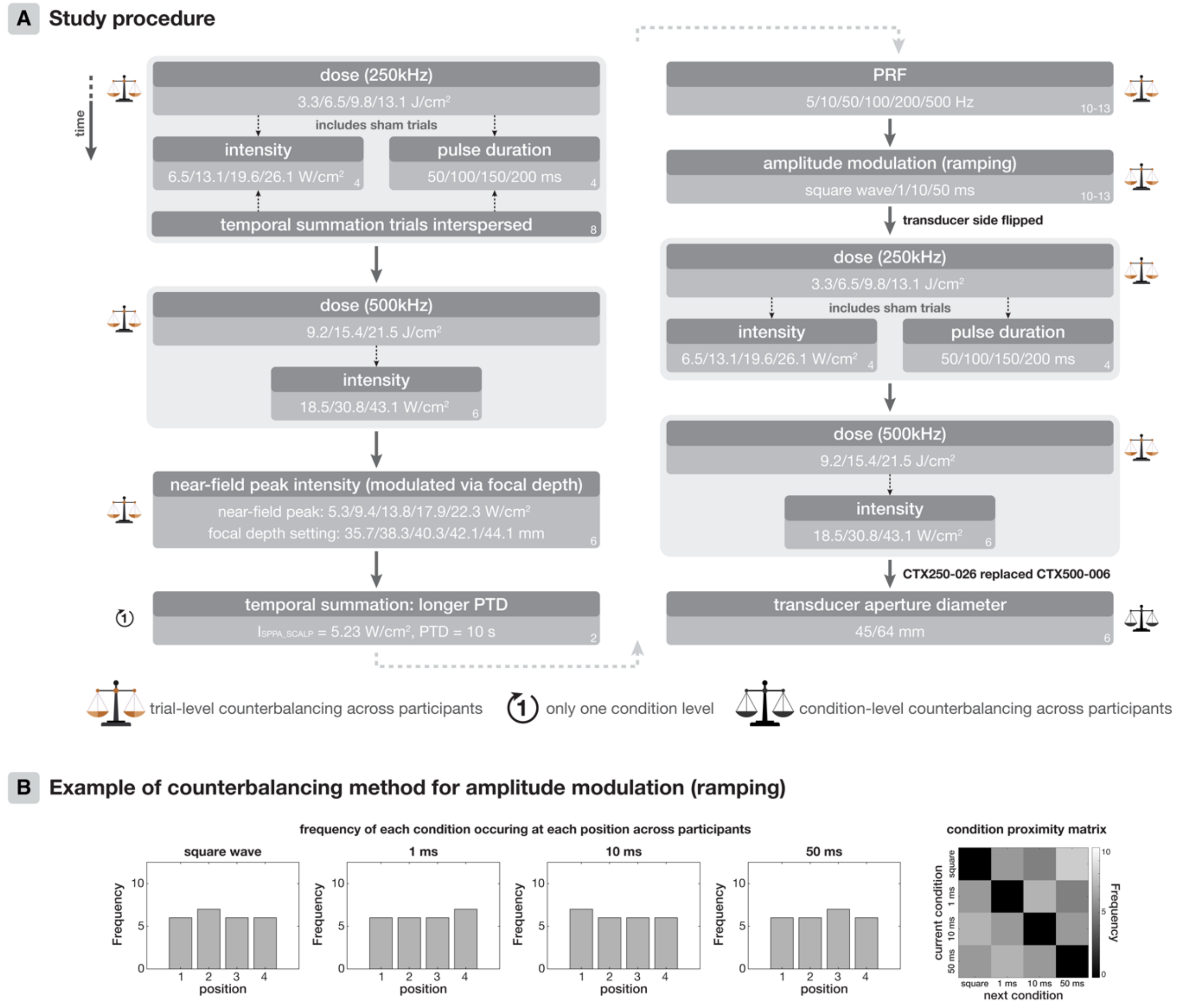

(A) Study procedure depicting the order of investigations taking place during the main experiment, with the type of counterbalancing indicated. **(B)** Counterbalancing example. We minimised variance in the frequency of conditions occurring across trial positions and the direct succession of conditions in one pair occurring more often than another pair. The frequency represents the number of participants in which that condition (e.g., square wave) occurs at each trial position.

At the beginning of the experiment, participants completed practice trials, including the lowest and highest doses for both transducers (250-2CH & 500-2CH), to familiarise themselves with VAS ratings and get an idea of what to expect during the experiment.

Next, different TUS parameters were manipulated in the order specified in Supplementary Fig. 5A. This uniform order was used so that any cumulative effects of stimulation across the experiment were equal for each investigated parameter, such that the total energy applied prior to each separate ‘research question’ was the same. To account for any possible interaction between temporal summation of peripheral somatosensation over time and differences in the initial side that 250 kHz and 500 kHz stimulation were applied, the starting side of the two transducers was also counterbalanced across participants.

bioRxiv preprint doi: https://doi.org/10.1101/2025.03.19.642045; this version posted March 19, 2025. The copyright holder for this preprint (which was not certified by peer review) is the author/funder, who has granted bioRxiv a license to display the preprint in perpetuity. It is made available under aCC-BY 4.0 International license.

Participants took a short break after the 'temporal summation: longer PTD' and 'amplitude modulation (ramping)' segments. Transducers were re-positioned and re-coupled at these times, as well as prior to the 'transducer aperture diameter' segment. Throughout the experiment, coupling quality was visually monitored at ~5-minute intervals.

For all manipulated stimulation parameters except for 'transducer aperture diameter', we implemented trial-level counterbalancing across participants. First, we generated all possible orders of unique condition levels using MATLAB 2019b for each of the following parameters: intensity (250 kHz), pulse duration (250 kHz), intensity (500 kHz), near-field peak intensity modulated via focal depth, PRF and ramping. We then identified the subset of N = 25 orders that would optimise trial-level counterbalancing per condition.

We evaluated counterbalancing quality via two metrics. First, we determined the frequency of each condition being administered at each trial position across participants, aiming to minimise variability in these frequencies to ensure that conditions were distributed as evenly as possible. Second, we determined how often two specific condition levels occurred consecutively, aiming to prevent specific pairs of conditions from occurring more often than other pairs.

Specifically, we compiled 1e9 random sets of N=25 condition orders for each manipulated parameter. From these, we selected the set with the lowest variance in condition frequency across participants and minimal variance in consecutive condition transitions (Supplementary Fig. 5B).

For VAS measurements, the same condition was repeated multiple times (*n*) per participant (see bottom-right of each box in Supplementary Fig. 5A). Here, the same order of conditions was presented *n* times. While this repetition could potentially amplify condition order effects within individuals, this approach mitigates the risk that temporal summation of somatosensory co-stimulation across trials could differentially impact different conditions within a single participant. Moreover, applying conditions in sets allowed for comparisons between successive sets to assess temporally summative effects across multiple protocols simultaneously (see main text Fig. 4D). By counterbalancing across participants, we have effectively controlled for condition order effects at the between-subject level.

The investigation of dose for 250 kHz stimulation included counterbalanced orders generated separately for 'intensity' and 'pulse duration' modalities, each including the sham condition as a level. This design resulted in 8 counterbalanced sham trials delivered to each side of the head. The orders for 'intensity' and 'pulse duration' dose modalities were then interleaved, with the starting modality counterbalanced between participants. Additionally, in the first 'dose' block we included interspersed trials with our standard protocol at ~1 minute intervals to monitor potential temporally summative effects on somatosensory co-stimulation across trials (see Supplementary Fig. 9).

To investigate the effects of transducer aperture diameter, we administered six consecutive trials each of the standard stimulation protocol using the two-element 250-2CH and four-element 250-4CH. Here, conditions were measured consecutively to capture any temporal summation of somatosensory co-stimulation for identical consecutive trials (see Supplementary

bioRxiv preprint doi: https://doi.org/10.1101/2025.03.19.642045; this version posted March 19, 2025. The copyright holder for this preprint (which was not certified by peer review) is the author/funder, who has granted bioRxiv a license to display the preprint in perpetuity. It is made available under aCC-BY 4.0 International license.

Fig. 9). Which transducer was tested first was counterbalanced across participants, as was the side of the transducers.

Sensory thresholds were measured for 'PRF' and 'ramping' sub-experiments. Here, 10-13 trials of the same protocol, administered at different intensities, were repeated to find the intensity at which the participant could perceive the stimulus 50% of the time (see Supplementary Fig. 4 for full details on the thresholding procedure).

Finally, to assess temporal summation of somatosensory co-stimulation during a longer PTD, mimicking the types of protocols applied in 'offline' TUS studies, we administered the standard protocol at an $I_{SPPA.SCALP}$ of 5.23 W/cm^2 for a 10 second PTD and participants continuously reported their sensations on a VAS. Participants practiced the continuous VAS scale once without TUS and then completed this procedure twice with 10 s PTD TUS.

bioRxiv preprint doi: https://doi.org/10.1101/2025.03.19.642045; this version posted March 19, 2025. The copyright holder for this preprint (which was not certified by peer review) is the author/funder, who has granted bioRxiv a license to display the preprint in perpetuity. It is made available under aCC-BY 4.0 International license.

Supplementary Fig. 6. | VAS responses to sham trials

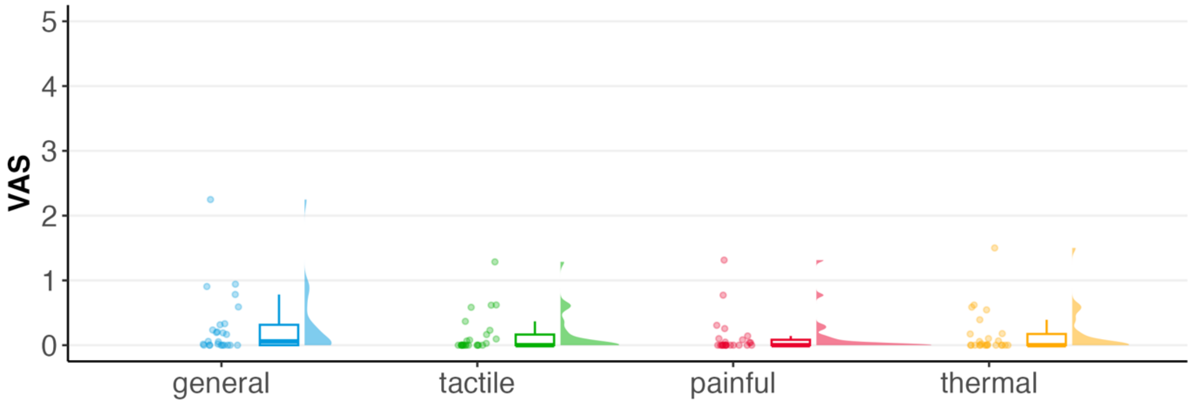

The sham condition, consisting of an auditory stimulus administered over speakers, elicited minor somatosensory effects in some participants. Points represent participant-level medians used for sham-correction. Boxplots and half-violins reflect the distribution of the data.

bioRxiv preprint doi: https://doi.org/10.1101/2025.03.19.642045; this version posted March 19, 2025. The copyright holder for this preprint (which was not certified by peer review) is the author/funder, who has granted bioRxiv a license to display the preprint in perpetuity. It is made available under aCC-BY 4.0 International license.

Supplementary Fig. 7 | Co-occurrence of somatosensory percepts

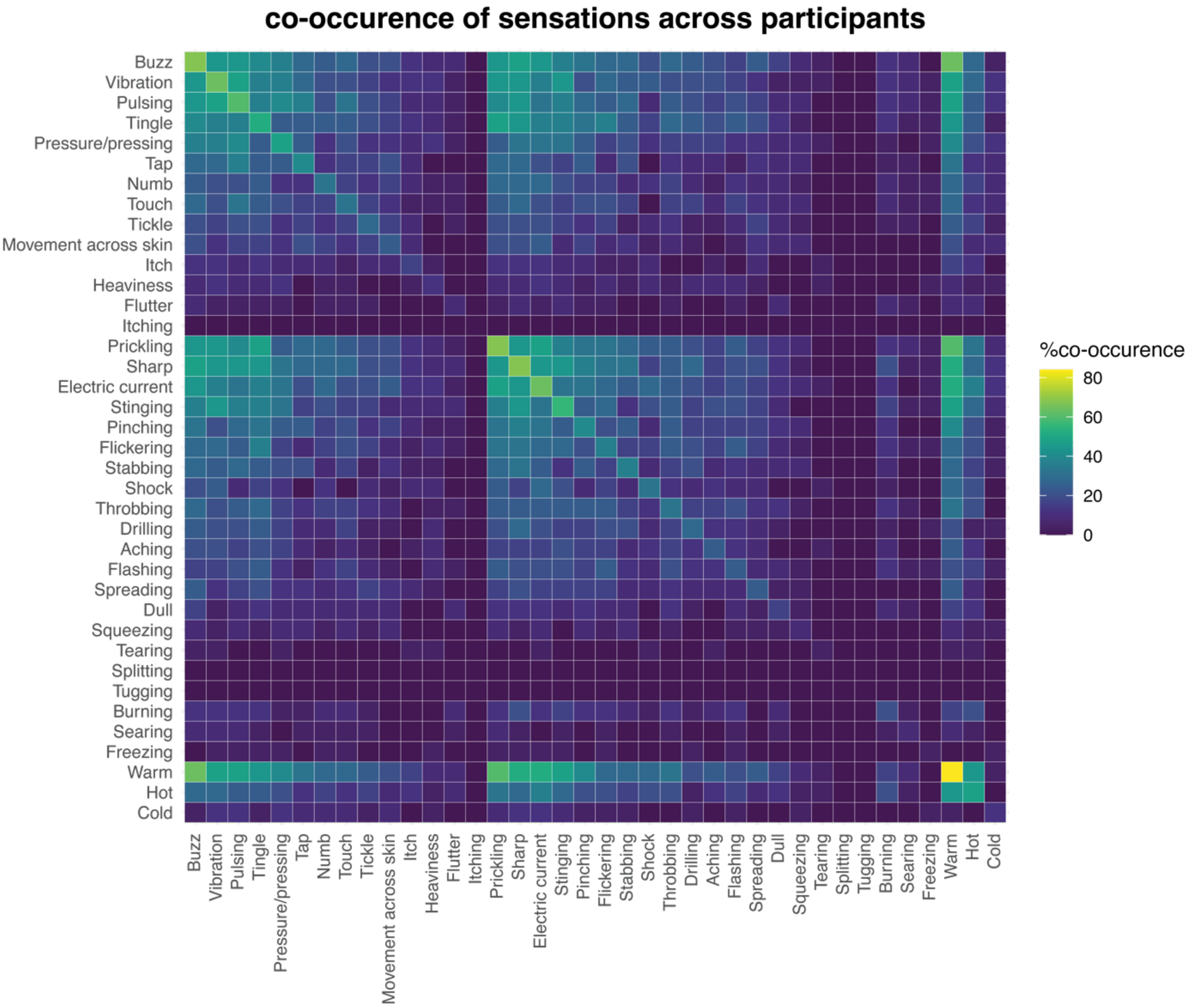

Co-occurrence of items on the psychometric questionnaire. Percentages reflect the proportion of participants that felt each pair of sensations. The diagonal depicts the percentage of participants that reported feeling each individual sensation.

bioRxiv preprint doi: https://doi.org/10.1101/2025.03.19.642045; this version posted March 19, 2025. The copyright holder for this preprint (which was not certified by peer review) is the author/funder, who has granted bioRxiv a license to display the preprint in perpetuity. It is made available under aCC-BY 4.0 International license.

Supplementary Fig. 8 | Dose-response (500 kHz)

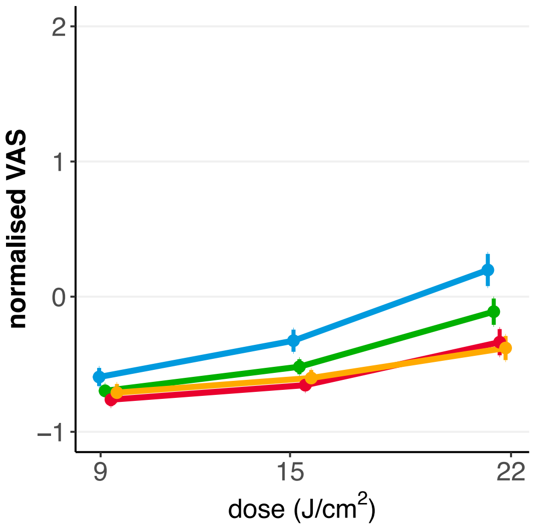

There was a significant effect of dose on VAS ratings for 500 kHz TUS. Points represent mean normalised VAS ratings across participants, and error bars depict standard error. Blue = general, green = tactile, orange = thermal, red = painful.

bioRxiv preprint doi: https://doi.org/10.1101/2025.03.19.642045; this version posted March 19, 2025. The copyright holder for this preprint (which was not certified by peer review) is the author/funder, who has granted bioRxiv a license to display the preprint in perpetuity. It is made available under aCC-BY 4.0 International license.

Supplementary Fig. 9 | Inter-trial temporal summation

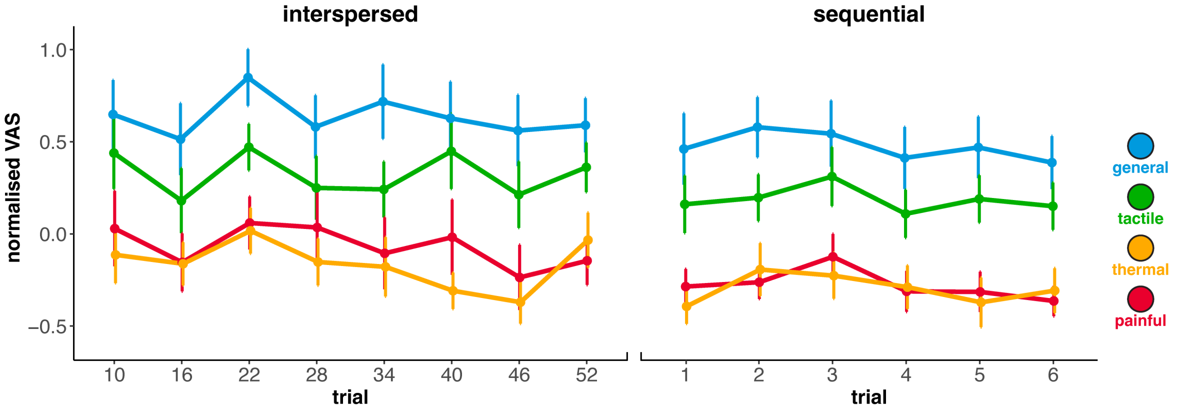

There was no significant effect of trial on VAS ratings, with Bayesian analyses providing strong evidence for the null hypothesis (see main text for statistics). This result holds both for identical trials delivered interspersed throughout a block (left) and delivered consecutively (right). Points depict mean normalised VAS ratings; error bars depict standard error.

bioRxiv preprint doi: https://doi.org/10.1101/2025.03.19.642045; this version posted March 19, 2025. The copyright holder for this preprint (which was not certified by peer review) is the author/funder, who has granted bioRxiv a license to display the preprint in perpetuity. It is made available under aCC-BY 4.0 International license.

References

1. Martin E, Aubry JF, Schafer M, Verhagen L, Treeby B, Pauly KB. ITRUSST Consensus on Standardised Reporting for Transcranial Ultrasound Stimulation. *ArXiv*. Published online February 15, 2024:arXiv:2402.10027v1.

2. Aubry JF, Attali D, Schafer M, et al. ITRUSST Consensus on Biophysical Safety for Transcranial Ultrasonic Stimulation. Published online July 12, 2024. doi:10.48550/arXiv.2311.05359

3. Haeussinger FB, Heinzel S, Hahn T, Schecklmann M, Ehlis AC, Fallgatter AJ. Simulation of Near-Infrared Light Absorption Considering Individual Head and Prefrontal Cortex Anatomy: Implications for Optical Neuroimaging. *PLOS ONE*. 2011;6(10):e26377. doi:10.1371/journal.pone.0026377

4. Light AE. Histological study of human scalps exhibiting various degrees of non-specific baldness. *J Invest Dermatol*. 1949;13(2):53-59. doi:10.1038/jid.1949.67

5. GARN SM, SELBY S, YOUNG R. SCALP THICKNESS AND THE FAT-LOSS THEORY OF BALDING. *AMA Archives of Dermatology and Syphilology*. 1954;70(5):601-608. doi:10.1001/archderm.1954.01540230051006

6. Treeby BE, Cox BT. k-Wave: MATLAB toolbox for the simulation and reconstruction of photoacoustic wave fields. *J Biomed Opt*. 2010;15(2):021314. doi:10.1117/1.3360308

7. Miscouridou M, Pineda-Pardo JA, Stagg CJ, Treeby BE, Stanziola A. Classical and Learned MR to Pseudo-CT Mappings for Accurate Transcranial Ultrasound Simulation. *IEEE Trans Ultrason Ferroelectr Freq Control*. 2022;69(10):2896-2905. doi:10.1109/TUFFC.2022.3198522

8. Leek MR. Adaptive procedures in psychophysical research. *Perception & Psychophysics*. 2001;63(8):1279-1292. doi:10.3758/BF03194543

9. Holmes NP, ed. *Somatosensory Research Methods*. Humana Press; 2023.

10. Taylor MM, Creelman CD. PEST: Efficient Estimates on Probability Functions. *The Journal of the Acoustical Society of America*. 1967;41(4A):782-787. doi:10.1121/1.1910407
